# Patient Selection for Epicardial Ablation—Part I: The Role of Epicardial Ablation in Various Cardiac Disease States

**DOI:** 10.19102/icrm.2019.101104

**Published:** 2019-11-15

**Authors:** Justin A. Edward, Duy T. Nguyen

**Affiliations:** ^1^Section of Cardiac Electrophysiology, Division of Cardiology, University of Colorado Denver, Aurora, CO, USA

**Keywords:** Arrhythmia, catheter, epicardial, radiofrequency ablation

## Abstract

Epicardial catheter ablation is most commonly performed following unsuccessful endocardial ablation. Given the frequency of epicardial substrates in certain cardiomyopathic disease states, however, a combined endocardial–epicardial approach should be considered as a primary treatment strategy. Although epicardial ablation is primarily deployed in patients with ventricular arrhythmias, the role of epicardial approaches in supraventricular tachycardias (eg, atrial fibrillation, inappropriate sinus tachycardia, and—rarely—accessory pathways) is growing, with continued advances being made.

## Introduction

Epicardial catheter ablation is most commonly relied on for the management of arrhythmogenic substrates arising outside of the endocardium. Most patients considered for epicardial ablation have already undergone an attempted endocardial catheter ablation. The sites of origin for various arrhythmias, particularly ventricular tachycardia (VT), are not limited to the subendocardial myocardium. In particular, patients with nonischemic cardiomyopathy can have either intramural or subepicardial substrates that give rise to VT. Epicardial catheter ablation for the treatment of VT is often required when critical elements of VT circuits are epicardial in origin.^1^ The technique of percutaneous epicardial mapping and ablation was first described in patients with Chagas cardiomyopathy as a means to ablate VT more than 20 years ago.^1^ Prior to the development of epicardial catheter ablation, patients with ventricular arrhythmias that were nonresponsive to endocardial ablation often required surgical intervention.

Similar advances have been made for patients experiencing symptomatic supraventricular tachycardias (SVTs), including drug-refractory atrial fibrillation (AF). Pulmonary vein (PV) isolation is recognized as the cornerstone of AF ablation.^2^ Previously, catheter ablation of AF has been limited to the right atrial (RA) and left atrial (LA) endocardia, and only surgical approaches have employed epicardial AF ablation access.^3,4^ In patients with recurrent AF despite PV isolation, additional arrhythmia sources outside of the PV antrum are suspected. More recently, hybrid procedures combining epicardial and endocardial ablation for AF have been utilized and have demonstrated better efficacy than isolated ablation procedures.^5^ This paper aims to review the indications, techniques, and developments in the field of epicardial catheter ablation as well as to highlight the unique subset of patients in whom epicardial ablation may be indicated.

## Indications for epicardial catheter ablation in ventricular tachycardia

Epicardial ablation is most commonly performed following unsuccessful endocardial ablation of midmyocardial or epicardial substrates. However, it may also be considered based on the etiology of the underlying disease process, substrate localization on imaging, specific electrocardiographic (ECG) criteria, or prior mapping demonstrating circuits of epicardial origin. In these cases, a combined endocardial–epicardial approach is associated with improved freedom from VT recurrence when compared with limited endocardial strategies.

### Epicardial ventricular tachycardia in various cardiomyopathic disease states

Although the use of epicardial ablation has previously been less common, recently, at high-volume centers, it is becoming a more routine procedure. Of the information available prior to VT ablation, the type of cardiomyopathy is probably the best predictor for determining the necessity of an epicardial approach. Epicardial ablation should be considered when there is a high pretest probability of midmyocardial or subepicardial scar, which is commonly seen in patients with idiopathic dilated cardiomyopathy (IDCM), arrhythmogenic right ventricular (RV) cardiomyopathy (ARVC), and hypertrophic cardiomyopathy (HCM). In particular types of cardiomyopathy, however, it is not only important to determine if epicardial ablation needs to be performed but to also identify when a combination of endocardial and epicardial ablation is warranted **(Table 1)**.

### Idiopathic dilated cardiomyopathy

The prevalence of epicardial VT in patients with nonischemic dilated cardiomyopathy is higher in comparison with in those with an ischemic VT substrate.^6,7^ In patients with IDCM, the main mechanism of VT is myocardial reentry. Pathological arrhythmogenic foci often originate at sites of scar tissue over the basal lateral left ventricle (LV) adjacent to mitral and aortic valve annuli deep in the endocardium.^6^ Areas of scar, however, may be larger in the epicardium than in the endocardium and, in some patients, are entirely limited to the epicardial surface, giving rise to arrhythmias that cannot be ablated via an endocardial approach. Contrast-enhanced cardiac magnetic resonance imaging (MRI) in patients with IDCM reveals midwall myocardial fibrosis in about one-third of cases, a finding that has been suggested as predictive of future arrhythmic events.^8^ Intracardiac echocardiography (ICE) has also been used to identify increased echogenicity in the lateral wall of the LV, which has been found to correspond with abnormal epicardial substrate and thereby guide an epicardial approach.^9^ Combined endocardial and epicardial catheter ablation in patients with IDCM and VT has achieved high rates of arrhythmia-free outcomes.^10,11^ Many patients in these studies had previously failed prior endocardial ablation. Thus, although choosing epicardial ablation prior to an endocardial approach in patients with IDCM may not always be the first-line option, epicardial ablation should be considered for all patients who previously failed endocardial ablation.

### Arrhythmogenic right ventricular cardiomyopathy

ARVC is an inherited disease characterized by fibrofatty tissue replacement of the myocytes predominantly in the RV. Early studies with endocardial ablation alone of VT in ARVC patients yielded suboptimal results, with almost half of patients experiencing arrhythmia recurrence despite taking antiarrhythmic drugs.^12^ An autopsy study of patients with ARVC found increased fibrofatty tissue deposition at the epicardial surface and, thus, epicardial circuits are generally indicated as the source of arrhythmia in these individuals.^13^ Accordingly, concomitant endocardial and epicardial mapping in ARVC patients revealed greater areas of low-voltage electrograms on the epicardial surface.^14^ The use of epicardial ablation as an adjunctive strategy with an endocardial approach has been reported in many observational studies of ARVC, and combined endocardial–epicardial mapping and ablation has been shown to reduce VT recurrence.^14–16^ A meta-analysis comparing combined endocardial–epicardial VT ablation and an endocardial-alone approach in ARVC patients found higher rates of freedom from recurrent ventricular arrhythmias or implantable cardioverter-defibrillator (ICD) placement with the former.^17^ Often, to prevent VT recurrence in ARVC patients, both endocardial and epicardial ablation should be performed.

### Chagas cardiomyopathy

The first described study of percutaneously accessing the pericardial space for epicardial VT ablation involved patients with Chagas cardiomyopathy.^1^ It was later reported that deploying combined endocardial–epicardial VT ablation in these patients could achieve higher arrhythmia-free success rates.^18^ Epicardial ablation is most likely beneficial in this specific group of patients because it has been shown that patients with Chagas cardiomyopathy and VT have an approximately twofold greater epicardial versus endocardial scar area.^19^ Due to the presence of subepicardial circuits, a combined endocardial–epicardial approach should be used in patients with Chagas cardiomyopathy in order to ablate reentrant circuits spanning both the subendocardium and epicardium.

### Viral myocarditis

Outside of the realm of Chagas myocarditis, there have been very few reported cases demonstrating the role of epicardial VT ablation for myocarditis. The application of contrast-enhanced cardiac MRI in patients with active myocarditis, however, has revealed inflammation present in the midwall and subepicardium, lending support to the notion that epicardial ablation may have an important role in ablating VT in myocarditis patients.^20,21^ In a study of 20 patients with drug-refractory VT and biopsy-proven viral myocarditis, approximately one-third of patients required epicardial ablation to successfully eliminate the arrhythmia.^22^ As viral myocarditis is often a self-limiting process, the current data do not support the role of epicardial ablation in all patients with myocarditis, as VT can often cease spontaneously during recovery.

### Cardiac sarcoidosis

Patients with cardiac sarcoidosis also boast a significant epicardial substrate with a predilection of granulomas in the subepicardium and interventricular septum.^23^ In a systematic review of five VT ablation studies in cardiac sarcoidosis patients, approximately 20% of the total 83 patients required epicardial ablation.^24^ VT burden is high in cardiac sarcoidosis patients, with a mean of three VTs per patient. It is therefore not surprising that the rate of freedom from VT recurrence was only 50% at a median follow-up point of 24 months, with about one-fourth of patients having to undergo redo procedures.^24,25^

### Hypertrophic cardiomyopathy

In HCM, monomorphic VT with an epicardial substrate may be present in up to 80% of affected patients.^26^ Although the predominant VT circuits are distributed in the basal septum and anterolateral LV and may be amenable to endocardial ablation, epicardial VT mapping and ablation should be considered in patients with refractory VT.^27,28^ Epicardial ablation in HCM is mostly pertinent for patients with apical aneurysms, which compose a minority of individuals with HCM. Most patients with septal hypertrophy have arrhythmias that are not approachable from the epicardium.

### Brugada syndrome

In contrast with other etiologies of cardiomyopathy, Brugada syndrome gives rise to ventricular arrhythmias in the epicardium of the anterior RV outflow tract, and epicardial ablation of this pathological substrate can render VT noninducible and provide long-term arrhythmia-free success.^29^ In patients with Brugada syndrome who underwent both endocardial and epicardial mapping, there was no identifiable endocardial substrate in 93% of cases, thus supporting the notion that an epicardial approach would favor freedom from recurrent ventricular arrhythmias.^30^

### Ischemic cardiomyopathy

In patients with ischemic cardiomyopathy, postinfarct scar is located in the subendocardium and can extend to the epicardium depending upon the patient’s specific coronary artery distribution. The arrhythmogenic tissue can be accessed via the endocardium, and it has been commonly thought that epicardial ablation strategies have lower yield in postinfarction patients.^31^ The prevalence of epicardial VT in patients with a history of myocardial infarction ranges from 14% to 33%.^32^ A recent study of patients with ischemic cardiomyopathy found abnormal epicardial substrates in 14% of patients.^33^ In a large series of patients with postinfarction VT, epicardial ablation was primarily performed after failed endocardial ablation and an epicardial approach was successful in abolishing the VT circuit in approximately 6% of the population.^34^ A combined endocardial–epicardial ablation strategy resulted in higher rates of VT-free survival after almost three years of follow-up as compared with endocardial ablation alone.^33^ In patients with ischemic cardiomyopathy, one must consider not only ablation history but also evidence of abnormal epicardial substrate with fractionated or split electrograms that may be visualized during epicardial mapping; these patients are those who may be ideal candidates for combined endocardial–epicardial ablation.

### Imaging modalities for epicardial arrhythmogenic substrates

An unsuccessful endocardial ablation can suggest that the offending substrate may not be accessible via an endocardial approach. In this subset of patients, preprocedural imaging can be particularly useful if it demonstrates or helps to identify epicardial substrates. As such, understanding the underlying arrhythmogenic substrate leading to VT is essential in achieving a successful ablation procedure.

Contrast-enhanced cardiac MRI is considered the gold standard to delineate three-dimensional scar tissue. The location of the scar on preprocedural MRI can help guide one to employ an endocardial and/or epicardial approach in order to reach the VT circuit. The location of scar may help to predict the location of a critical VT isthmus.^35^ In patients with nonischemic cardiomyopathy, scar tissue is most commonly located either intramurally or on the epicardial surface. However, the lowest rates of successful ablation have been reported in patients in whom the majority of scar had an intramural distribution.^35^ In patients with ARVC, the electrical substrate most often originates in the epicardium, likely because the disease process begins at this myocardial surface. MRI can be integrated with electroanatomical mapping to provide higher resolution for the detection and localization of nontransmural scar as compared with other imaging modalities.^36^ It must be noted, however, that the most important limitation of MRI is its use in cardiac device recipients, as the majority of patients undergoing VT ablation also have an ICD. Although ICDs may not necessarily preclude MRI given recent studies and improved MRI compatbility,^37,38^ imaging of the heart may be impaired due to artifacts created by the pulse generator or lead/coil.^39^

ICE provides real-time, two-dimensional anatomical information and allows for the monitoring of catheter–tissue contact, lesion formation, and potential complications without the risk of radiation exposure from other imaging methods.^40^ In addition, it may have a role in identifying epicardial scar as VT substrate. In a study of patients with nonischemic cardiomyopathy with recurrent VT and abnormal echogenicity on ICE imaging, it was demonstrated that areas of increased echogenicity in the lateral wall corresponded with the abnormal epicardial substrate seen on electroanatomic mapping when reviewed by a blinded echocardiographer.^9^ These abnormal areas of increased echogenicity were absent in the control group, who had structurally normal hearts.

### Role of electrocardiogram criteria in epicardial ventricular tachycardia

ECG criteria may be clinically useful when assessing for the presence of epicardial substrate. Several groups have used 12-lead ECG tracings to investigate specific characteristics that may be helpful prior to the electrophysiological study in determining the epicardial origin of VT. One of the first studies, published by Berruezo et al., suggested the following three ECG criteria are able to identify the epicardial origin of VT with good sensitivity and specificity: a pseudodelta wave of 34 ms or more (sensitivity: 83%; specificity: 95%), an intrinsicoid deflection time of 85 ms or more (sensitivity: 87%; specificity: 90%), and an R/S complex duration of 121 ms or more (sensitivity: 76%; specificity: 85%).^41^

A subsequent study by Bazan et al. suggested different site-specific ECG criteria for LV VT based on the initial vector of the QRS complex.^42^ Specifically, Bazan et al. found that the ECG criteria proposed by Berruezo et al. had varying degrees of specificity and sensitivity depending not only on the type of structural heart disease but also on different sites within the LV, which can vary in terms of local circuit activation. Site-specific morphology criteria support that the following must be present for epicardial LV VT: a Q-wave in lead I for basal superior and apical superior sites, the absence of a Q-wave in any of the inferior leads for basal superior sites, and a Q-wave in inferior leads for basal inferior and apical inferior sites. These proposed morphology criteria correctly identified epicardial origin in 84% of VT cases.^42^ Based on prior studies, Vallès et al. proposed an algorithm to identify epicardial VT originating from the basal superior and lateral LV in patients with nonischemic cardiomyopathy.^43^ The absence of a Q-wave in the inferior leads with either a pseudo-delta wave of more than 75 ms, a maximum deflection index of more than 0.59, and/or the presence of a Q-wave in lead I are indicative of epicardial origin with both a sensitivity and specificity of more than 90%.^43^

These criteria have proven to be optimal parameters to guide the recognition of an epicardial source primarily in patients with nonischemic cardiomyopathy.

ECG criteria can be helpful in determining potential circuits of epicardial VT origin; however, one must take into account the type of cardiomyopathy and the imaging of potential VT substrate when applying the previously mentioned ECG criteria in the assessment of VT.

### Endocardial mapping

ECG criteria are not always reliable in diagnosing epicardial VT and, in some cases, detailed endocardial mapping can be predictive of epicardial VT circuits **(Table 2)**. In one particular study of patients with VT who were referred for ablation, bipolar electrograms (EGMs) in areas of early endocardial activation were compared with areas of early epicardial activation using electroanatomic activation mapping.^44^ Three characteristics were found from endocardial mapping that consistently indicated the need for epicardial ablation: (1) diffuse early activation (> 2 cm^2^ region of sites equally having their earliest activation within 10 ms); (2) the sequence of a far-field EGM followed by a near-field EGM in the region of earliest endocardial activation; and (3) the inability to capture the far-field component of the earliest EGM (stim–QRS interval < EGM–QRS time) or reproduce morphological features of the ventricular arrhythmia complex with stimulation at the earliest endocardial site of activation.

In patients with nonischemic cardiomyopathy and VT, endocardial unipolar low-voltage areas were effective in identifying regions with epicardial bipolar low voltages in 60% of cases.^45^ One study confirmed that low endocardial unipolar voltages of below 4.0 mV are independent predictors of epicardial bipolar scar with 80% accuracy (and 71% sensitivity and 75% specificity).^46^ Endocardial mapping has also been found to be predictive of epicardial VT substrates in patients with both ARVC and ischemic cardiomyopathy.^33,47^

## Epicardial catheter ablation in supraventricular tachycardias

An epicardial approach can also be used for SVT ablation; however, it is most commonly employed following unsuccessful endocardial ablation.

### Accessory pathways

Epicardial pathway circuits have been implicated in up to 8% of failed endocardial ablations of accessory pathways.^48^ In a series of 10 patients with accessory pathways and previously failed endocardial ablations, the earliest activation was recorded epicardially in five cases with a variety of accessory pathway locations including the right atrial appendage to right ventricle, left posteroseptal region, and right posterolateral region.^49^ At two years of follow-up, one patient showed a recurrence of arrhythmia and was successfully reablated. Additional small cases series have been reported of successful epicardial ablation of accessory pathways following failed initial endocardial ablation.^50,51^ In a separate study of 21 patients referred for accessory pathway epicardial ablation after a median of two prior failed endocardial procedures, almost 30% of the patients had evidence of early epicardial activation.^52^ Extensive epicardial mapping followed by a combined endocardial–epicardial ablation procedure was effective in eliminating the accessory pathway. In patients who failed to show a benefit after endocardial ablation for accessory pathways, epicardial mapping followed by simultaneous endocardal–epicardial ablation should be considered.^53^

### Atrial fibrillation

As is true with epicardial ablation for VT, epicardial catheter ablation in AF is often attempted after failed endocardial PV isolation. Surgical approaches such as the Cox maze procedure were first introduced more than 25 years ago to treat patients with refractory AF.^54^ Although successful in reducing the recurrence of AF, this surgical procedure requires completion of a sternotomy. Over the last 15 years, there has been a push toward minimally invasive procedures and the introduction of thorascopic approaches has led to the replacement of prior cut-and-sew lesions.^55^ Surgical ablation is often used to treat AF in patients in whom medications and catheter ablation have failed. Currently, the two primary types of surgical ablation are convergent (or hybrid) ablation and Cox maze IV ablation. The Cox maze IV procedure, first developed in 2002, is a modification from prior procedures in that it incorporates bipolar radiofrequency ablation and cryoablation. However, it still requires sternotomy and the continued need for cardiopulmonary bypass. This procedure had one-year success rates approaching 90%, with more than half of patients presenting with either persistent or long-standing AF.^56^ Five-year outcomes following the Cox maze IV procedure suggest rates of 78% and 66% for overall freedom from AF and freedom from AF while off antiarrhythmic drugs, respectively.^57^ A single-center, propensity-matched study reported 10-year survival rates in patients following Cox maze IV and those with untreated AF of 62% and 42%, respectively.^58^

In a collaboration between cardiothoracic surgeons and electrophysiologists, percutaneous endocardial catheter mapping and ablation were first added to epicardial thorascopic ablation with the hope of increasing the success rates by combining potential advantages of each separate procedure.^59^ This hybrid AF procedure allowed for fast epicardial placement of extensive linear lesions with the endovascular validation of complete bidirectional conduction block. The convergent ablation procedure is different from previous hybrid ablations in that it employs a novel device to perform epicardial ablation via a transdiaphragmatic approach. Single-center studies over the past 10 years have noted success rates of greater than 80% in restoring sinus rhythm in patients with persistent AF.^60–62^ Furthermore, among patients followed for up to four years after the procedure, sinus rhythm was maintained in more than 80% of cases.^63,64^ However, although efficacious in restoring sinus rhythm, a recent meta-analysis of prior studies raises concerns about the use of the convergent procedure due to a complication rate of 9% and a procedural mortality rate of 1.7%.^65^

Isolated epicardial ablation for recurrent atrial arrhythmias has been shown to give rise to iatrogenic circuits with predisposition to atrial flutter.^5,66^ This potential risk of arrhythmias has been thought to be due to the limitations of current ablation techniques in creating transmural lesion sets when applied endoscopically on the epicardium of the beating heart.^67^ The ideal ablation strategy for recurrent AF would be to create durable transmural lesion sets in a minimally invasive fashion as well as to map out other potential arrhythmogenic foci, thus reducing the possible risk of arrhythmia recurrence.^68^ More recently, however, a combined endocardial and subxiphoid epicardial catheter approach has been used to both map and ablate recurrent AF.^69^ Potential advantages of this new approach include that it is less invasive than thorascopic epicardial ablation and it also provides transmural ablation lines while mapping potential additional arrhythmia mechanisms. These recent studies suggest that, perhaps, with appropriate patient selection and the accurate identification of patient-specific mechanisms, this novel approach may provide an advantage over contemporary techniques with regard to improving patient outcomes after AF ablation and preventing the recurrence of atrial arrhythmias.

### Inappropriate sinus tachycardia

**Figure 1:**
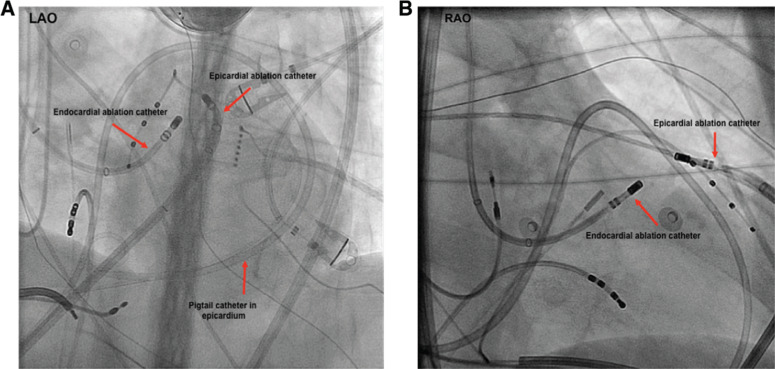
Endocardial–epicardial bipolar ablation configuration. Bipolar ablation across an LV summit midmyocardial circuit may require epicardial access for one of the ablation poles. **A and B:** Fluoroscopic views (anteroposterior) of two ablation catheters in a bipolar configuration, targeting the LV summit area for this patient. LAO: left anterior oblique; RAO: right anterior oblique.

Successful epicardial ablation has also been reported for inappropriate sinus tachycardia (IST). Sinus node modification with endocardial catheter ablation for the treatment of IST remains a challenge due to the location of the phrenic nerve. While this concern may be greater when employing an epicardial approach given the proximity of the phrenic nerve to the epicardial surface of the heart, the epicardial approach allows for mechanical displacement of the phrenic nerve and therefore safer delivery of energy to the sinus node either epicardially or endocardially **(Figure 1)**.^70^

There are some adverse effects that can present with sinus node ablation, including right diaphragmatic paralysis, transient superior vena cava syndrome, and complete sinus node dysfunction requiring pacing.^71^ More recently, in a case series of five patients who had previously failed endocardial ablation, combined endocardial–epicardial sinus node ablation was found to be successful in resolving symptoms, although three patients did develop postoperative pericarditis.^72^ Several nonrandomized studies have assessed the efficacy of radiofrequency catheter ablation with varying degrees of success.^49,73–75^ A meta-analysis of nine prior ablation studies highlighted acute procedural success rates approaching 90%; however, only one study utilizing an epicardial approach was included.^76^ In addition, 8.5% of patients in this study had severe procedural complications (eg, diaphragmatic paralysis, arteriovenous fistula, retroperitoneal bleed) and almost 10% of patients required pacemaker implantation.^76^ Additional studies need to be performed to determine the safety and efficacy of endocardial–epicardial ablation in patients with refractory IST.^77^ In many cases of IST, the risks of ablation may outweigh the benefits, and the procedure is not currently recommended in the guidelines as first-line therapy.

## Conclusion

Many factors play a role in the selection of patients for epicardial mapping and ablation as well as in the safe and effective delivery of ablation lesions in the epicardium; above, we have touched upon a few of the criteria and ablation parameters in this review. Understanding the VT substrate is important in those with nonischemic cardiomyopathy because the epicardium is commonly the site of arrhythmogenic foci. Furthermore, the role of an epicardial approach in patients with SVT is ever-expanding with the novel technological advances ongoing in catheter ablation.

## Figures and Tables

**Table 1:** Prevalence of Epicardial VT Origins in Various Cardiomyopathic Disease States

Type of Cardiomyopathy	Potential Site(s) of Epicardial Circuits	Role of Epicardial Ablation	Frequency of Epicardial Substrate
Idiopathic dilated cardiomyopathy	Scar tissue over basal lateral LV	Epicardial ablation should be considered in all patients who have previously failed endocardial ablation	High
Arrhythmogenic right ventricular cardiomyopathy	Fibrofatty tissue deposition at the epicardial surface	Combined endocardial–epicardial approach should be considered as first-line therapy; performance of ablation early on in disease course leads to lower recurrence rates of VT	High
Chagas cardiomyopathy	Predominant scar spanning both the subendocardium and epicardium	Combined endocardial–epicardial approach is the first-line therapy and should be considered to ablate subendocardial and epicardial circuits	High
Viral myocarditis	Inflammation in the midwall and subepicardium	Epicardial ablation is not the first-line therapy; however, some patients may have epicardial circuits	Low
Cardiac sarcoidosis	Granulomas in the subepicardium and interventricular septum	Epicardial ablation should be considered in all patients	High
Hypertrophic cardiomyopathy	Basal septum and anterolateral LV	Predominant VT circuits are amenable to endocardial ablation; however, an epicardial substrate is common in monomorphic VT	Moderate to high
Brugada syndrome	Epicardium of the anterior right ventricular outflow tract	Epicardial ablation should be performed in all patients	High
Ischemic cardiomyopathy	Subendocardial postinfarct scar can extend to epicardium based on coronary artery lesion distribution	Combined endocardial–epicardial approach may have clinical utility and may be considered based on clinical features and endocardial mapping	Low to moderate

LV: left ventricle; VT: ventricular tachycardia.

**Table 2:** Clinical Features Suggestive of Epicardial VT Origins

Underlying Substrate	ECG Findings	Imaging	Endocardial Mapping
Cardiomyopathies (as listed in **Table 1**)	NICM: • Absence of inferior Q-waves • Pseudo-delta wave > 75 ms • MDI > 0.59 • Presence of Q-wave in lead I	Subepicardial or midmyocardial scar detected either on contrast-enhanced computed tomography or magnetic resonance imaging	• Diffuse early activation (>2 cm^2^ sites in 10 ms) • Far-field early EGM followed by near-field EGM • Inability to capture far-field component of earliest EGM • Unipolar voltage suggestive of epicardial scar
	ICM: • Pseudo-delta wave > 34 ms • IDT > 85 ms • Shortest RS complex > 121 ms • QRS duration > 211 ms		

ECG: electrocardiogram; EGM: electrogram; IDT: intrinsicoid deflection time; MDI: maximum deflection index.
